# Temporal and Spatial Dynamics of Inflammasome Activation After Ischemic Stroke

**DOI:** 10.3389/fneur.2021.621555

**Published:** 2021-04-22

**Authors:** Danli Lu, Mengyan Hu, Bingjun Zhang, Yinyao Lin, Qiang Zhu, Xuejiao Men, Zhengqi Lu, Wei Cai

**Affiliations:** ^1^Department of Neurology, Mental and Neurological Disease Research Center, The Third Affiliated Hospital of Sun Yat-sen University, Guangzhou, China; ^2^Center of Clinical Immunology, Mental and Neurological Disease Research Center, The Third Affiliated Hospital of Sun Yat-sen University, Guangzhou, China

**Keywords:** brain ischemia, inflammasome, macrophages, neuroprotection, outcome

## Abstract

**Background:** The inflammasome represents a highly pro-inflammatory mechanism. It has been identified that inflammasome was activated after ischemic stroke. However, the impact of inflammasomes on stroke outcomes remains contradictory. The participating molecules and the functioning arena of post-stroke inflammasome activation are still elusive.

**Methods:** In the present study, blood samples from stroke patients were collected and analyzed with flow cytometry to evaluate the correlation of inflammasome activation and stroke outcomes. A stroke model was established using male C57/Bl6 mice with transient middle cerebral artery occlusion (tMCAO, 1 h). The dynamics of inflammasome components, cell type, and location of inflammasome activation and the therapeutic effects of inhibiting post-stroke inflammasome executors were evaluated.

**Results:** We found that a high level of inflammasome activation might indicate detrimental stroke outcomes in patients and mice models. Post-stroke inflammasome activation, especially NLRP3, cleaved Caspase-1, cleaved Caspase-11, IL-1β, IL-18, and GSDMD, peaked at 3–5 days and declined at 7 days with the participation of multiple components in mice. Macrophage that infiltrated into the ischemic lesion was the main arena for post-stroke inflammasome activation among myeloid cells according to the data of mice. Among all the members of the Caspase family, Caspase-1 and −11 served as the main executing enzymes. Inhibiting Caspase-1/−11 signaling efficiently suppressed DAMPs-induced macrophage inflammasome activation and displayed neuroprotection to stroke models including infarct size (Control: 48.05 ± 14.98; Cas1.i: 19.34 ± 12.21; Cas11.i: 21.43 ± 14.67, *P* < 0.001) and neurological deficit score (0 d-Control: 2.20 ± 0.63; 0 d-Cas1.i: 2.20 ± 0.63; 0 d-Cas11.i: 2.20 ± 0.63; 1 d-Control: 2.50 ± 0.53; 1 d-Cas1.i: 1.50 ± 0.71; 1 d-Cas11.i: 2.00 ± 0.67; 2 d-Control: 2.30 ± 0.48; 2 d-Cas1.i: 1.30 ± 0.48; 2 d-Cas11.i: 1.50 ± 0.53; 3 d-Control: 2.00 ± 0.67; 3 d-Cas1.i: 1.20 ± 0.42; 3 d-Cas11.i: 1.30 ± 0.48, *P* < 0.001).

**Conclusions:** Taken together, inflammasome activation played a detrimental role in stroke pathology. Targeting post-stroke inflammasome executing enzymes fitting in the dynamics of macrophages might obtain potential and efficient therapeutic effects.

## Introduction

Activation of inflammasomes is one of the key mechanisms leading to inflammatory processes. It has been found to exert non-substitutable functions during infection (1). Nevertheless, in the context of aseptic diseases, such as auto-immune disorders, activation of inflammasomes causes unfavorable injuries (2). Inflammasome formation has been identified after acute ischemic stroke (AIS) (3, 4). However, the impact of inflammasomes on stroke outcomes remains elusive. On one hand, suppression of inflammasomes has been demonstrated to protect against ischemic stroke concomitant with diabetes (5). On the other hand, the involvement of inflammasome activation in the secondary infarct extension was doubted (6). Inflammasomes involve multiple components including different kinds of sensors [the nucleotide-binding oligomerization domain (NOD), leucine-rich repeat (LRR)-containing protein (NLR) family members (NLRP1, NLRP2, and NLRP3), NLR family-caspase recruitment domain (CARD)-containing protein 4 (NLRC4), and absent in melanoma 2 (AIM2)], executors (Caspase-1, Caspase-8, and Caspase-11), and substrates [interleukin-1β (IL-1β), IL-18, and Gasdermin-D (GSDMD)]. IL-1β and IL-18 can be cleaved by Caspase-1 to the active form as pro-inflammation molecules. Both Caspase-1 and Caspase-11 could activate GSDMD, which resulted in a specific cell death process called pyroptosis (7, 8).

Among all the components of inflammasomes, the dispatchers that take part in the process of inflammasome activation vary according to the disease settings (9, 10). Components that participate in post-stroke inflammasome activation are to be explored. Inflammasome formation has been found in diverse cell kinds including dendritic cells, neutrophils, and macrophages (11–13), but we still need to determine which cell type the inflammasome mainly activates after a stroke. The executors in acute ischemic stroke remain elusive. Therefore, the current study first identified the main participants of inflammasome activation in stroke models and then explored the therapeutic effects targeting the identified targets.

In this study, we aim to affirm the therapeutic value of inflammasome suppression, identifying the participants in post-stroke inflammasome activation and the arena of inflammasome formation. The protective effects of targeting inflammasome executing enzymes were also explored.

## Materials and Methods

### Ethical Statement

The clinical and the animal experimental studies were approved by the Medical Ethics Committee of the Third Affiliated Hospital of Sun Yat-Sen University and the Animal Care and Use Committee of Sun Yat-Sen University, respectively. All participants gave their informed consent according to the principles illustrated in the Declaration of Helsinki.

### Patients

In this study, 26 stroke patients recruited in the Third Affiliated Hospital of Sun Yat-Sen University from July 2018 to October 2019 consecutively had an independently documented primary stroke event in combination with confirmed magnetic resonance imaging (MRI) evidence showing ischemic stroke. The inclusion and exclusion criteria have been published previously (14). Age (mean 64 and 95% CI 52–75), gender (female:male = 9:17), and scores on the National Institute stroke scale (NIHSS) of patients were recorded. A total of 40 age-matched healthy community dwellings was recruited as healthy controls (HCs). Patient demographics including co-morbidities were summarized in Table 1.

**Table 1:** Demographics of the included patients and healthy controls (HC).

**Variables (*N*)**	**HC (40)**	**Patients (26)**
Females, *N* (%)	13 (33)	9 (31)
Age, y, median (IQR)	60 (52–68)	64 (52–75)
**Risk factors**, *N* **(%)**		
Hypertension	-	13 (50)
Diabetes mellitus	-	5 (19)
Heart failure	-	0 (0)
Coronary disease	-	3 (12)
Atrial fibrillation	-	3 (12)
Smoking	-	6 (23)
Alcohol consumption	-	5 (19)
NIHSS scale on admission, median (IQR)	-	5 (2–7)
NIHSS scale on discharge, median (IQR)	-	2 (0.75–5)
Infarct size on admission, median (IQR, cm)	-	2.48 (1.02–3.91)

*IQR, interquartile range; NIHSS, NIH Stroke Scale; NLR, neutrophil lymphocyte ratio; HC, Healthy controls*.

### MRI Scanning and Infarct Volume Analysis of Patients

Magnetic resonance imaging (MRI) was performed within 24 h of admission using 1.5- or 3.0-T magnetic resonance imaging (Signa; GE Medical Systems, Milwaukee, WI, USA). In this study, the diffusion-weighted imaging (DWI) spin-echo planar sequence included 20 contiguous axial oblique slices (b = 0 and 1000 s/mm^2^ isotropically weighted; repetition time/echo time, 6000/60.4 ms; acquisition matrix, 128 × 128; slice thickness, 5 mm; interslice gap, 1 mm; field of view, 24 cm). DWI lesions in 26 patients were measured with Analyze 7.0 software (Analyze Direct, KS). Cerebral infarct sizes were assessed by the largest infarct diameter determined on the image demonstrating the largest lesion (15). MRI scans of patients were assessed by experienced neurologist Zhengqi Lu, who was blinded to the patients' clinical features. All images were interpreted with the same window settings, same types of monitors, and lighting conditions.

### Animals

C57/Bl6 wild type mice (age 8–12 weeks and weight 18–25 g) were purchased from Guangdong Medical Laboratory Animal Center (Guangzhou, China) and housed in a humidity- and temperature-controlled animal facility in Sun Yat-sen University with a 12-h light–dark cycle for at least 1 week before induction of ischemic stroke. Food and water were freely accessible. All the experimental protocols were approved by the Animal Care and Use Committee of Sun Yat-sen University following the Guide for the Care and Use of Laboratory Animals. A total of 71 male mice were used in the transient middle cerebral artery occlusion (tMCAO, 1 h) model, including 11 mice that were excluded from further assessments due to death after ischemia or unsuccessful induction of stroke. There were 18 male mice used for primary macrophage-enriched culture.

### Animal Experiments Statement

The animal research has been written up in accordance with the ARRIVE (Animal Research: Reporting *in vivo* Experiments) guidelines.

### Murine Model of Transient Focal Ischemia

A focal ischemic stroke model was induced in mice with transient middle cerebral artery occlusion (tMCAO) as previously described (16). Briefly, mice were anesthetized with 1.5% isoflurane in the air under spontaneous breathing. A midline neck incision was made, and soft tissues were retracted gently over the trachea. The left common carotid artery (CCA) was isolated and ligated temporarily. A permanent knot was placed on the distal part of the external carotid artery (ECA), and a loose temporal knot was placed on both the proximal part of ECA. A tight temporal knot was placed on the internal carotid artery (ICA). ECA was cut between the permanent knot and the temporal knot, and a filament was inserted into ECA and directed to the middle cerebral artery (MCA) through ICA after loosening the temporal knot on ICA. Once the filament insertion into the MCA was confirmed, the loose temporal knot on ECA was tightened and the temporal knot on CCA was loosened. Filament insertion into the ICA was maintained for 60 min and followed by reperfusion. Sham-operated animals underwent the same anesthesia and surgical procedures but were not subjected to arterial occlusion. Core body temperatures were maintained with a heating pad. Regional cerebral blood flow (rCBF) during the surgery was measured by laser Doppler flowmetry. Mice with <70% reduction of blood flow in the ischemic core or those that died during surgery were excluded from further analysis.

### Flow Cytometric Analysis

Blood samples were collected from AIS patients and healthy controls. A lysis buffer (Roche) was applied to samples for 10 min to exclude red blood cells (RBC). Single cells then were stained with surface markers of CD11c-PE (1:400, Biolegend), CD11b-PE-Cy7 (1:400, Biolegend), CD66b-PerCP-Cy5.5 (1:400, Biolegend), and CD14-APC-Cy7 (1:400, Biolegend). Cells were then fixed and permeabilized. Antibodies of anti-NLRP3 (Rabbit, abcam) and anti-IL1β (goat, R&D system) were then applied to cells overnight. After washing, cells were incubated with secondary antibodies of anti-rabbit-Alexa Flour 488 and anti-goat-Alexa Flour 488 for 30 min. Cells were then subjected to analysis after wash.

Blood and brain were extracted from animals at indicated time points. Brains were dissected, and ipsilateral hemispheres were collected after saline perfusion. Each hemisphere was subjected to 0.25% trypsin-EDTA (Thermo Fisher) digestion at 37°C for 25 min. Brain tissue was then pressed through a cell strainer (70 μm, Thermo Fisher). Brain cells were separated from myelin debris by centrifugation in 30/70% Percoll solution (GE healthcare). Brain cells at the interface were collected, washed with PBS, and subjected to further staining. Red blood cell lysis buffer (Sigma Aldrich) was applied to get rid of red blood cells in the blood that were subjected to further staining.

Single cells from brain and blood were stained with CD45-PerCp/Cy5.5 (1:400, Biolegend), CD11b-PE (1:400, Biolegend), CD11c-APC/Cy7 (1:400, Biolegend), and F4/80-BV421(1:400, Biolegend). Cells were then fixed and permeabilized. Antibodies of anti-NLRP3 (Rabbit, abcam) and anti-IL1β (goat, R&D system) were then applied to cells overnight. After washing, cells were incubated with secondary antibodies of anti-goat-Alexa Flour 488 and anti-rabbit-BV421 for 30 min. Cells were then subjected to analysis after wash.

Flow cytometric analysis was performed using a fluorescence-activated cell sorter flow cytometer (BD Biosciences), and data were analyzed using FlowJo X 10.0.7r2 software.

### Western Blot

Mice were sacrificed at indicated time points after tMCAO with CO_2_. After perfusion with iced saline, ipsilateral brains were extracted and homogenized with 1 ml of lysis buffer (Sigma Aldrich) with a protease inhibitor (Roche), phosphatase inhibitor (Roche), and PMSF (Sigma Aldrich). Samples were then centrifuged (12,000G, 20 min), and the supernatant was collected. Western blots were performed using the standard SDS-polyacrylamide gel electrophoresis method and enhanced chemiluminescence detection reagents (Thermo Scientific). Antibodies against NLRP3 (Cell signaling technology, 1:1000), NLRC4 (Cell signaling technology, 1:1000), AIM2 (Cell signaling technology, 1:1000), Caspase-1 (Santacruz, 1:500), Caspase-8 (Cell signaling technology, 1:1000), Caspase-11 (Abcam, 1:500), IL-1β (R&D system, 1:500), IL-18 (Cell signaling technology, 1:1000), GSDMD (Abcam, 1:500), and GAPDH (Cell signaling technology, 1:3000) were used. Immunoreactivity was semi-quantitatively analyzed by Image J (NIH). Images of blots were cropped for presentation.

### Primary Macrophage-Enriched Culture

Primary macrophage-enriched cultures were prepared from the bone marrow of 8- to 10-week-old C57/Bl6 mice. Monocytes were isolated using a mouse monocyte isolation kit (STEMCELL Technologies) according to the manufacturer's instructions. Monocytes were then cultured in the presence of macrophage colony-stimulating factor (MCSF, 20 ng/ml) for 7 days *in vitro* to induce macrophage.

### Preparation of Danger Associated Molecular Patterns (DAMPs)

DAMPs were collected from the ipsilateral brain 3 days after ischemic stroke using methods previously described (17). Briefly, mice were sacrificed and perfused with sterile iced saline. The ipsilateral brain was extracted and digested with 2 ml of 0.25% Trypsin for 20 min at 37°C. For each hemisphere, 5 ml of RPMI1640 containing 10% FBS was applied to stop digestion. Samples were then centrifuged, and the supernatant was collected. The supernatant was then pressed first through a 0.22 μm filter to exclude any cell component. The solution of DAMPs were then treated to macrophage for inflammasome induction.

### Inhibitor of Inflammasome

For macrophage, the concentration of Caspase-1 inhibitor (Belnacasan, MCE) was 1 μM (18), and Caspase-11 inhibitor (Wedelolactone, MCE) was 5 μM (19). As for the mice, an inhibitor of Caspase-1 or Caspase-11 was applied to them (10 mg/kg i.v.) (20) at 1 h after reperfusion via the caudal vein.

### Statistical Analysis

Results were presented as mean ± *standard deviation* (*SD*). GraphPad Prism software (version 8.0; La Jolla, CA) was used for statistical analyses. The two-tailed *Student's t test* was used for the comparison of the two groups. The differences in means among multiple groups were analyzed using *one-way analysis of variance* (*ANOVA*) for continuous variables with normal distribution. Linear regression was used to analyze the correlation between two sets of continuous variables. When *ANOVA* showed significant differences, pair-wise comparisons between means were tested by *post hoc* Bonferroni tests. In all analyses, *P* ≤ 0.05 was considered statistically significant.

## Results

### High Level of Inflammasome Product Indicates Detrimental Stroke Outcomes

**Figure 1:**
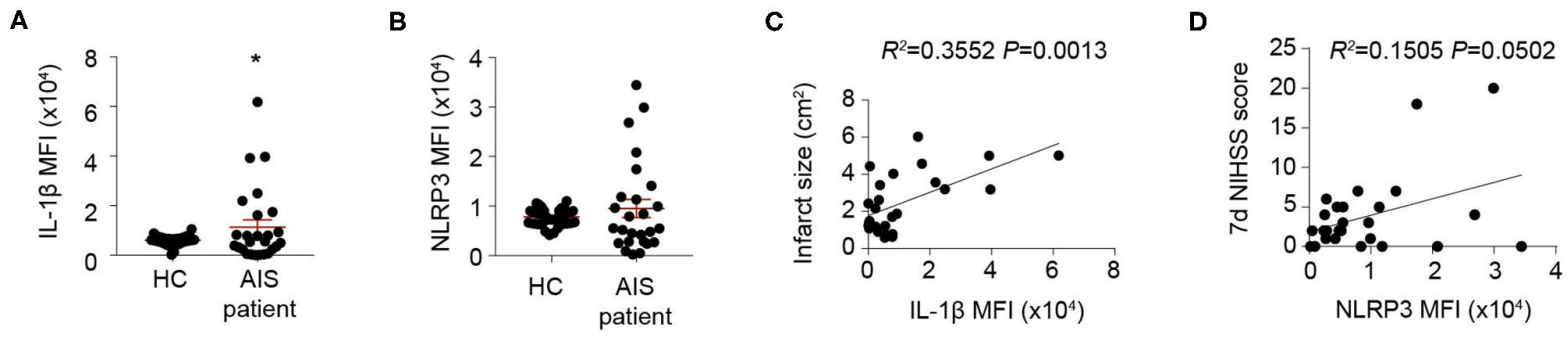
Inflammasomes are activated and indicates detrimental stroke outcome. **(A,B**) Peripheral blood samples were collected from 40 healthy controls (HC) and 26 patients suffering an acute ischemic stroke (AIS) within the first 24 h of symptom onset. Inflammasome markers IL-1β **(A)** and NLRP3 **(B)** were analyzed with flow cytometry. *P < 0.05 vs. HC in *Student t-test*. **(C,D)** Mean fluorescent intensity (MFI) of IL-1β was calculated and correlation of IL-1β MFI with infarct size was analyzed with *Linear regression* **(C)**, and MFI of NLRP3 was calculated and correlation of NLRP3 MFI with NIHSS score at 7 d after disease onset **(D)** was analyzed with *Linear regression*.

To confirm the impact of inflammasome activation on stroke outcomes, blood samples from 26 stroke patients and 40 sex- and age-matched healthy controls (Table 1) were collected and inflammasome markers NLRP3 and IL-1β were analyzed. As is reported, the level of IL-1β (inflammasome product) increased at 1 day after AIS (HC: 6,064.85 ± 1,735.25; AIS patients: 1,1345.56 ± 1,5130.49, *P* < 0.05) (Figure 1A). Expression of NLRP3 (receptor of inflammasome) increased in a part of patients (Figure 1B). Nevertheless, it is not a significant result. Regression analysis revealed that Mean fluorescent intensity (MFI) of IL-1β (1 day) positively correlated with infarct size (*R*^2^ = 0.3552, *P* = 0.0013) (Figure 1C). MFI of NLRP3 (1 day) correlated somewhat with the 7-day NIHSS score (*R*^2^ = 0.1505, *P* = 0.0502) (Figure 1D). The results indicated that inflammasome activation may be correlated with detrimental stroke outcomes, indicating the rationale for studying post-stroke inflammasome activation as a therapeutic target.

### Dynamics of Inflammasome Formation After Ischemic Stroke

**Figure 2:**
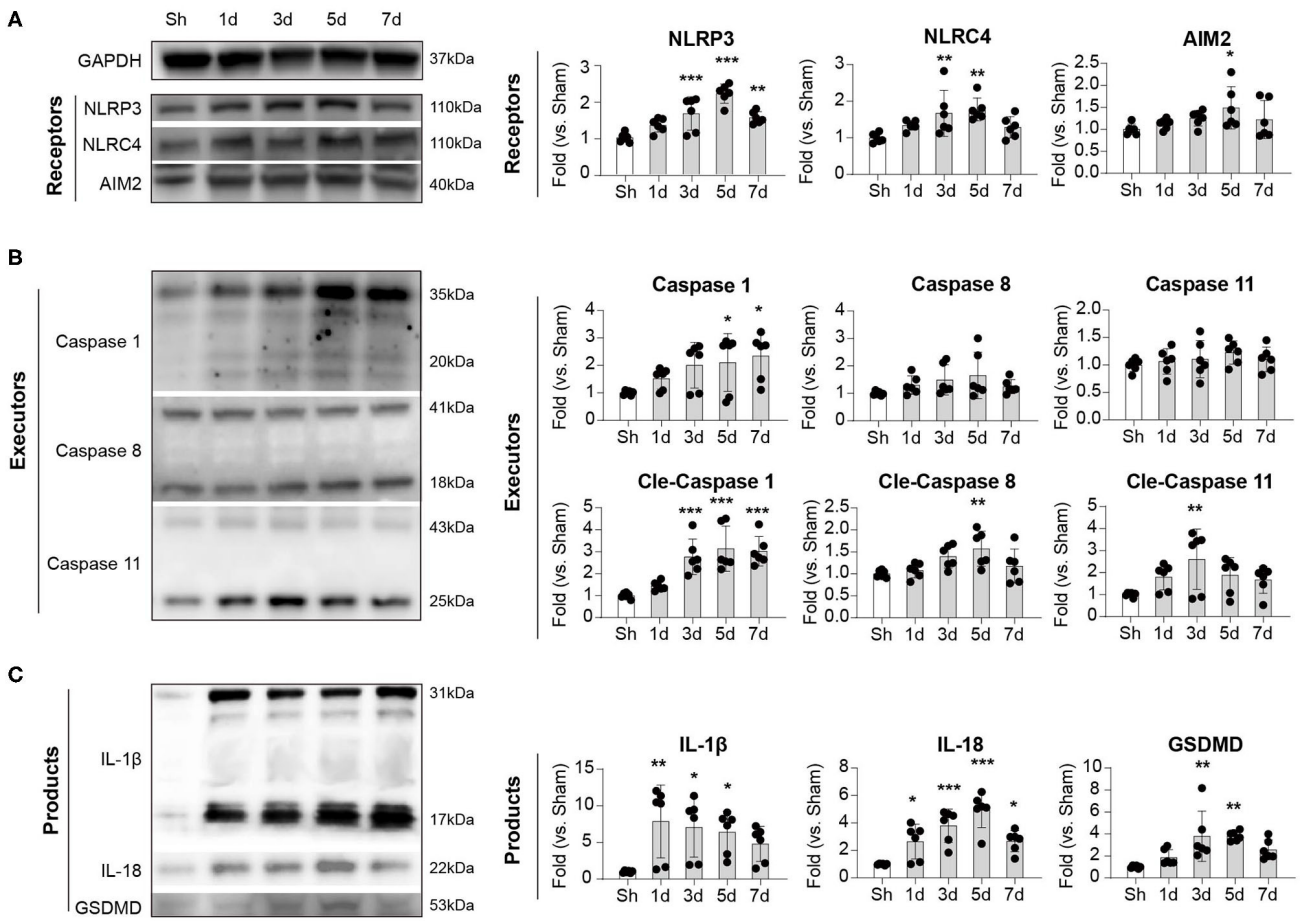
Dynamic alternation of **(A)** receptors, **(B)** executors, and **(C)** products of inflammasome after ischemic stroke. WT C57/Bl6 mice were subjected to Sham or tMCAO operation. Mice were sacrificed at indicated time points and whose brain tissues of the ipsilateral hemisphere were collected. Expression dynamics of inflammasome receptors (NLRP3, NLRC4, and AIM2), executors (Caspase 1, Caspase 8, and Caspase 11), and products (IL-1β, IL-18, and GSDMD) were analyzed by western blotting. Data were normalized to the expression of GAPDH. *N* = 4 at each time point. *P < 0.05, **P < 0.01, ***P < 0.001 vs. Sham (Sh) mice in *One-way ANOVA*.

We next explored the dynamics of inflammasome activation within the lesion of ischemic stroke. Ipsilateral brains of mice were collected at indicated time points, and inflammasome sensors (NLRP3, NLRC4, and AIM2), executors (Caspase-1,−8, and−11), and products (IL-1β, IL-18, and GSDMD) were analyzed with Western Blot. Expression of NLRP3, NLRC4, and AIM2 displayed similar dynamics after stroke, which increased as soon as 1 day, peaked at 3–5 days, then decreased by day 7. In particular, the dynamics of NLRP3 is the most obvious (Sh: 1.00 ± 0.12; 1 d: 1.34 ± 0.19; 3 d: 1.66 ± 0.46; 5 d: 2.21 ± 0.26; 7 d: 1.55 ± 0.16, *P* < 0.001) (Figure 2A). For the executors of inflammasome, both the complete and the functioning cleaved form of Caspase-1 continuously increased from day 1 to at least day 7, and the level of cleaved Caspase-1 changed clearly (Sh: 1.00 ± 0.13; 1 d: 1.44 ± 0.20; 3 d: 2.77 ± 0.81; 5 d: 3.14 ± 1.03; 7 d: 3.02 ± 0.67, *P* < 0.001). The cleaved Caspase-11 displayed similar dynamic (Sh: 1.00 ± 0.10; 1 d: 1.80 ± 0.59; 3 d: 2.61 ± 1.38; 5 d: 1.88 ± 0.81; 7 d: 1.67 ± 0.61, *P* < 0.05) as the inflammasome receptors (NLRP3, NLRC4 and AIM2), though the level of complete Caspase-11 remained steady. The cleaved Caspase-8 peaked slightly at day 5 after stroke (Sh: 1.00 ± 0.08; 1 d: 1.08 ± 0.17; 3 d: 1.39 ± 0.26; 5 d: 1.57 ± 0.40; 7 d: 1.18 ± 0.39, *P* < 0.05), while the level of complete Caspase-8 was also steady (Figure 2B). As for the inflammasome products, the activated cleaved IL-1β increased soon after stroke (1 day) (Sh: 1.00 ± 0.16; 1 d: 7.86 ± 4.98; 3 d: 7.07 ± 4.08; 5 d: 6.41 ± 2.84; 7 d: 4.79 ± 2.39, *P* < 0.05) and remained at high level until at least day 7. The expression dynamics of cleaved IL-18 (Sh: 1.00 ± 0.05; 1 d: 2.65 ± 1.26; 3 d: 3.80 ± 1.21; 5 d: 4.96 ± 1.29; 7 d: 2.68 ± 0.77, *P* < 0.001) and GSDMD (Sh: 1.00 ± 0.12; 1 d: 1.86 ± 0.71; 3 d: 3.79 ± 2.29; 5 d: 3.82 ± 0.43; 7 d: 2.57 ± 0.89, *P* < 0.001) resembled that of NLRP3, NLRC4, AIM2, cleaved Caspase-11 and cleaved Caspase-8 (Figure 2C). Conclusively, we showed that the receptors of NLRP3, NLRC4, and AIM2, the executors of Caspase-1 and−11 were the main contributors of-post stroke inflammasome activation, which participated in the production of IL-1β, IL-18, and GSDMD. According to the dynamics of various inflammasome components, activation of inflammasome was initiated timely and peaked at day 3–5 after ischemic stroke.

### Inflammasome Activated Most Robustly in Macrophage Among Infiltrated Myeloid Cells After Stroke

**Figure 3:**
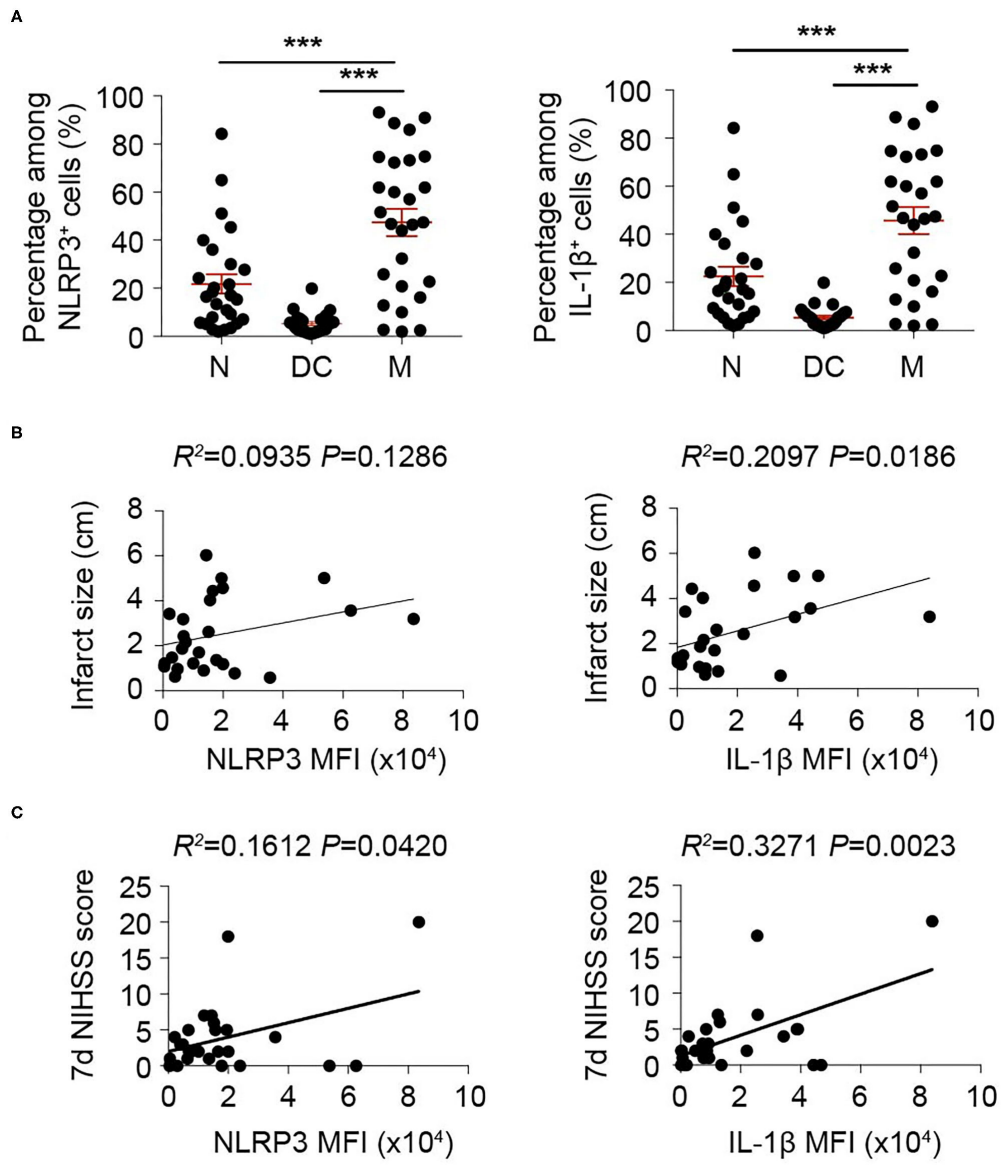
Inflammasome activation in monocyte/macrophage is correlated with adverse stroke severity. Peripheral blood from 26 patients with acute ischemic stroke was collected within 24 h of disease onset and analyzed with flow cytometry. **(A)** Cells that expressed NLRP3 and IL-1β were gated in flow cytometric analysis (FACS). Percentage in NLRP3^+^ or IL-1β^+^ cells of neutrophil (N, CD45^+^CD11b^+^CD66b^+^), dendritic cells (DC, CD45^+^CD11c^+^) and monocyte/macrophage (M, CD45^+^CD11b^+^CD14^+^) were assessed. ***P < 0.001 in *One-way ANOVA*. **(B,C)** Mean fluorescent intensity (MFI) of NLRP3 or IL-1β in monocyte/macrophage (CD45^+^CD11b^+^CD14^+^) was calculated. Correlation of NLRP3 MFI or IL-1β MFI with infarct size **(B)** or NIHSS score assessed at 7 d **(C)** after disease onset was analyzed with *Linear regression*.

**Figure 4:**
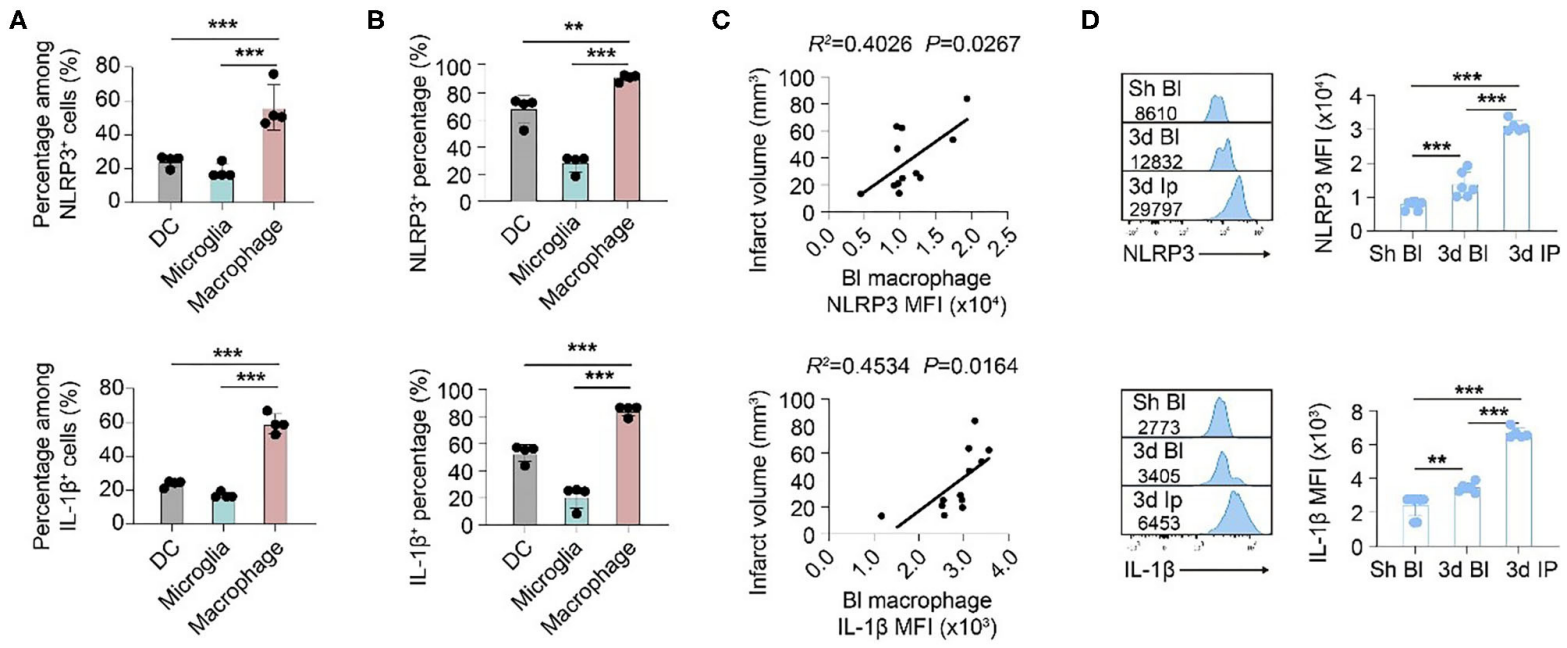
Inflammasomes activate primarily in macrophages that infiltrate into ischemic lesions after stroke. WT C57/Bl6 mice were subjected to Sham operation or tMCAO. Mice were sacrificed at 3 d after tMCAO when inflammasome activation peaked. **(A)** Brain cells that expressed NLRP3 and IL-1β were gated in flow cytometric analysis (FACS). Percentage in NLRP3^+^ or IL-1β^+^ cells of dendritic cells (DC, CD45^+^CD11c^+^), microglia (CD45^int^CD11b^+^), and macrophage (CD45^hi^CD11b^+^F4/80^+^) was assessed. *N* = 4 mice. ***P < 0.001 in *One-way ANOVA*. **(B)** Percentage of NLRP3 and IL-1β in dendritic cells (DC, CD45^+^CD11c^+^), microglia (CD45^int^CD11b^+^), and macrophage (CD45^hi^CD11b^+^F4/80^+^) in ipsilateral brain was calculated with flow cytometry. *N* = 4 mice. ***P < 0.001 in *One-way ANOVA*. **(C)** Mean fluorescent intensity (MFI) of NLRP3 or IL-1β in macrophages (CD45^hi^CD11b^+^F4/80^+^) in the peripheral blood of mice was calculated. Correlation of NLRP MFI or IL-1β MFI with infarct volume of mice at 3 d after stroke was analyzed with *Linear regression*. *N* = 10 mice. **(D)** NLRP MFI or IL-1β MFI in macrophage (CD45^hi^CD11b^+^F4/80^+^) in peripheral blood (Bl) of Sham (Sh) operated mice, peripheral blood of mice at 3 d after tMCAO and ipsilateral (Ip) brain was analyzed with flow cytometry. *N* = 4 mice. **P < 0.01, ***P < 0.001 in *One-way ANOVA*.

We then oriented the inflammasome-forming cell for better inflammasome control. Since inflammasomes are mainly activated in myeloid cells traditionally among current research (21) [only that neutrophil has limited inflammasome activation in mice (22)], we analyzed the inflammasome markers of NLRP3 and IL-1β in these cells. In both patients (Figure 3) and mice (Figure 4), macrophagess displayed the most robust inflammasome activation. At 1 day after stroke, macrophages took up the highest part of NLRP3^+^ (N: 15.26 ± 13.31%; DC: 4.75 ± 4.90%; M: 38.74 ± 25.65%, *P* < 0.001) or IL-1β^+^ (N: 21.80 ± 20.52; DC: 5.27 ± 4.13; M: 47.36 ± 29.30, *P* < 0.001) cells in patients' blood (Figure 3A). Accordingly, NLRP3- or IL1β-MFI of blood macrophage (1 day) positively correlated with patients' infarct volume (NLRP3: *R*^2^ = 0.0935, *P* = 0.1286; IL1β: *R*^2^= 0.2097, *P* = 0.0186) (Figure 3B) and/or NIHSS score (day 7) (NLRP3: *R*^2^ = 0.1612, *P* = 0.0420; IL1β: *R*^2^ = 0.3271, *P* = 0.0023) (Figure 3C). In the ischemic lesion of mice at day 3 after stroke (peak of inflammasome activation), over half of the NLRP3^+^ (DC: 24.55 ± 3.45%; Microglia: 18.43 ± 4.25%; Macrophage: 56.23 ± 13.33%, *P* < 0.001) or IL-1β^+^ (DC: 23.85 ± 1.92%; Microglia: 17.03 ± 1.65%; Macrophage: 59.35 ± 5.83%, *P* < 0.001) cells were macrophages (Figure 4A). After gating on macrophage, 91.13 ± 2.58% of macrophages were NLRP3^+^ (DC: 67.68 ± 10.28%; Microglia: 28.03 ± 6.36%; Macrophage: 91.13 ± 2.58%, *P* < 0.001), and 84.58 ± 3.92% were IL-1β^+^ (DC: 53.13 ± 6.32%; Microglia: 21.09 ± 8.58%; Macrophage: 84.58 ± 3.92%, *P* < 0.001) (Figure 4B). Similarly, NLRP3- (*R*^2^ = 0.4026, *P* = 0.0267) or IL1β-MFI (*R*^2^ = 0.4534, *P* = 0.0164) of blood macrophage (day 3) positively correlated with mice' infarct volume (Figure 4C). We next sought to identify the arena for inflammasome activation of macrophage. Compared with macrophage in Sham blood, NLRP3- (Sh Bl: 7843.50 ± 1219.12; 3 d Bl: 11769.61 ± 6224.95; 3 d Ip: 31003.80 ± 1760.71, *P* < 0.001) and IL1β-MFI (Sh Bl: 2420.50 ± 627.24; 3 d Bl: 6236.33 ± 4232.41; 3 d Ip: 6665.20 ± 311.82, *P* < 0.001) of blood macrophage at 3 days after stroke significantly elevated, which further increased when macrophage arrived at the stroke lesion (Ipsilateral brain) (Figure 4D), which indicated that inflammasome activation in macrophage began in peripheral blood but busted in the stroke lesion.

### Suppressing Caspase-1/11 Signaling to Inhibit Inflammasome Activation Protects Against Ischemic Stroke

**Figure 5:**
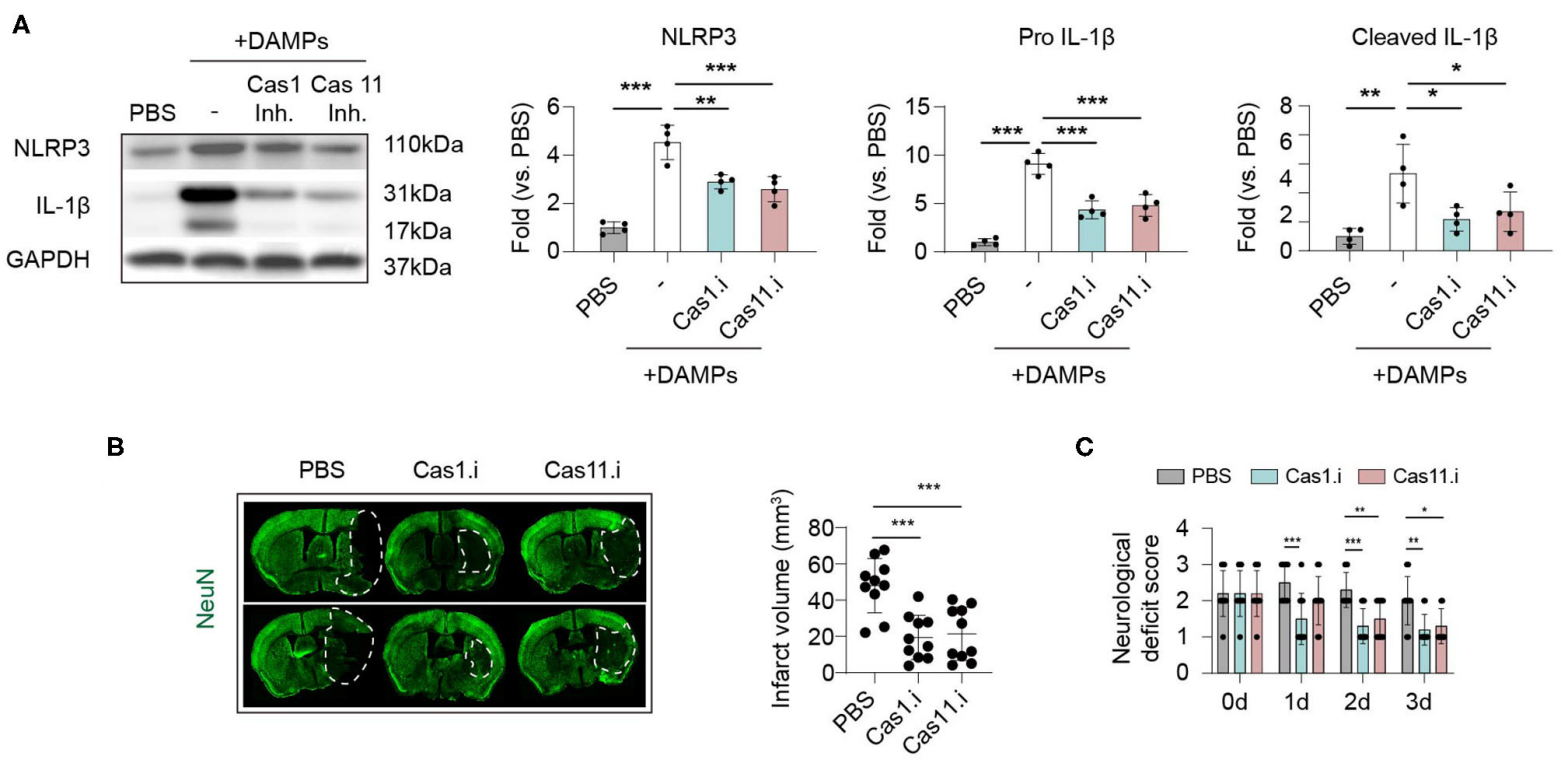
Inhibiting Caspase 1/11 signaling hinders inflammasome activation induced by brain ischemia and protects against ischemic stroke. **(A)** Primary cultured bone-marrow-derived macrophages were treated with Caspase 1 inhibitor Belnacasan (1 μM) or Caspase 11 inhibitor Wedelolactone (5 μM) overnight before inflammasome induction. Danger-associated molecular patterns (DAMPs) were prepared from the ipsilateral brain of mice at 3 d after tMCAO and treated to macrophages for 2 h. Inflammasome markers of NLRP3 and IL-1β were analyzed with western blotting. Data were normalized to the gray scale of GAPDH. Experiments were repeated three times. *P < 0.05, **P < 0.01, ***P < 0.001 in *One-way ANOVA*. **(B,C)** Wild-type C57/Bl6 mice were subjected to tMCAO and Caspase 1 inhibitor Belnacasan (10 mg/kg) or Caspase 11 inhibitor Wedelolactone (10 mg/kg), or equal volume of PBS was treated to mice at 1 h after reperfusion. *N* = 10 mice in each group. *P < 0.05, **P < 0.01 in *One-way ANOVA*. **(B)** Mice were sacrificed at 3 d after tMCAO, and infarct volume was assessed with immunostaining of NeuN. **(C)** Neurological deficit score was assessed from 0 to 3 d after tMCAO. *N* = 10 mice in each group. *P < 0.05, **P < 0.01 in *Two-way ANOVA*.

We have found that Caspase-1 and−11 were the main executors of post-stroke inflammasomes, and inflammasomes were activated mainly in macrophages within stroke lesions. Therefore, we explored whether suppressing Caspase-1 or−11 signaling could inhibit inflammasome activation in macrophages and its therapeutic effects in ischemic stroke. To mimic *in situ* pathophysiology, we extracted DAMPs (Danger associated molecular patterns) from the ipsilateral brain according to a previous study (17) then treated to macrophage for 2 h (Figure 5A). Strikingly, administration of brain derived DAMPS dramatically increased protein expression of NLPR3 and IL-1β (both complete and cleaved form) in macrophage compared with controls (treated with PBS), while application of Caspase-1 inhibitor (Belnacasan, 1 μM) or Caspase-11 inhibitor (Wedelolactone, 5 μM) significantly reversed the process (NLRP3: PBS: 1.00 ± 0.24; DAMPs: 4.53 ± 0.71; Cas1.i: 2.90 ± 0.30; Cas11.i: 2.59 ± 0.52, *P* < 0.001) (Pro IL-1β: PBS: 1.00 ± 0.36; DAMPs: 9.10 ± 1.07; Cas1.i: 4.35 ± 0.93; Cas11.i: 4.80 ± 1.12, *P* < 0.001) (Cleaved IL-1β: PBS: 1.00 ± 0.55; DAMPs: 5.33 ± 2.02; Cas1.i: 2.18 ± 0.81; Cas11.i: 2.70 ± 1.36, *P* < 0.01). To evaluate the therapeutic value of Caspase-1/-11 inhibition, AIS was induced in wild-type C57/Bl6 male mice. According to previous studies, infiltration of myeloid cells was initiated within hours of ischemic stroke (14, 23). On the other hand, our data indicated that activation of inflammasome could be pre-primed when cells were delivered to the lesion through peripheral blood. Therefore, inhibitors of Caspase-1 or Caspase-11 were injected intravenously and at 1 h, a time point prior to the pre-prime of inflammasome and the infiltration of myeloid cells into stroke lesion. Inhibitor of Caspase-1 or Caspase-11 was applied to mice (10 mg/kg i.v.) at 1 h after reperfusion, and both treatments, especially inhibitor of Caspase-1, showed favorable protection after stroke for infarct volume (PBS: 48.05 mm^3^ ± 14.98 mm^3^; Cas1.i: 19.34 mm^3^ ± 12.21 mm^3^; Cas11.i: 21.43 mm^3^ ± 14.67 mm^3^, *P* < 0.001) (Figure 5B) and neurological deficit score (0 d-PBS: 2.20 ± 0.63; 0 d-Cas1.i: 2.20 ± 0.63; 0 d-Cas11.i: 2.20 ± 0.63; 1 d-PBS: 2.50 ± 0.53; 1 d-Cas1.i: 1.50 ± 0.71; 1 d-Cas11.i: 2.00 ± 0.67; 2 d-PBS: 2.30 ± 0.48; 2 d-Cas1.i: 1.30 ± 0.48; 2 d-Cas11.i: 1.50 ± 0.53; 3 d-PBS: 2.00 ± 0.67; 3 d-Cas1.i: 1.20 ± 0.42; 3 d-Cas11.i: 1.30 ± 0.48, *P* < 0.001) (Figure 5C).

## Discussion

It is widely accepted that post-stroke inflammation promotes secondary tissue damage in the ischemic brain. Inflammasome activation represents a potent pro-inflammatory mechanism. The current study confirmed the occurrence of inflammasome activation after stroke and increased infarct volume. The dynamics of inflammasome components and the arena of inflammasome activation have been identified. Therapeutic effects of suppressing the main executors (Caspase-1 and−11) of post-stroke inflammasome activation was also explored.

The potential of targeting inflammasome in stroke therapy has been studied since its discovery. Among inflammasome components, NLRP3 is the most understood especially in aseptic inflammation. Mounting evidence has indicated that the NLRP3 inflammasome played a prominent role in the pathogenesis and progression of ischemic stroke (4). Inhibiting the NLRP3 inflammasome could be a promising therapeutic strategy (5, 24). However, contradictory ideas exist. Lemarchand, E. and colleagues reported that NLRP3 did not contribute to the extension of ischemic brain injury after ischemic stroke, as no protection to stroke was observed when applying NLRP3 inhibitor or knocking out NLRP3 in mice (6). We showed that the inflammasome product IL-1β elevated after AIS in both patients and mice models, which indicated the occurrence of inflammasome activation. Nevertheless, no expression alteration of NLRP3 level was detected in patients, which revealed that the function of NLRP3 might be negligible in post-stroke inflammasome activation. Indeed, the data on human specimens may not be directly paralleled with those in mice, since they have been obtained only in the blood of patients. Moreover, we found that besides NLRP3, other receptors, such as NLRC4, participated in post-stroke inflammasome formation in mice models, which indicates that NLRP3 is not the only path for inflammasome activation.

Our results revealed that post-stroke inflammasome activation involved multi-players, including various receptors, executors, and subsequent products. Among all the components, executing enzymes play a check-point-like role. Whichever receptors detect the dangerous signals would activate the downstream executing enzymes for further procession. It seems that the executors of inflammasome vary depending on the disease pathology (9, 10). It has been reported that it was Caspase-8 that played a decisive role in multiple sclerosis (10), whereas our data suggested that it was Caspase-1/-11 that represented the primary executors in post-stroke inflammasome activation. Since Caspase-1 and −11 keep the gate of post-stroke inflammasome activation, targeting the enzymes for inflammasome inhibition may be more efficient than the receptors. Indeed, treatment of Caspase-1/-11 inhibitor after ischemic stroke offered favorable neuroprotection.

Inflammasome activates primarily in myeloid cells. On the other hand, several kinds of brain cells have been shown to display inflammasome formation (e.g., neuron, astrocyte, and endothelial cells) (11–13). Our results suggested that it was macrophages that endured the most robust inflammasome activation after stroke among myeloid cells since over half of the inflammasome activating cells in the stroke brain were macrophages. Moreover, inflammasome activation in macrophages occurred primarily within the stroke lesion. Orienting the cell type and location of inflammasome activation provides clues for the improvement of therapeutic efficiency. However, we lack the data on the stroke lesion in patients, which needs further improvement. Our data suggested that fitting the time point for macrophage infiltration and increasing BBB entry of inflammasome suppressors may favor their therapeutic effects.

In conclusion, we found that inflammasome activated mainly in macrophages that infiltrated into stroke lesion and played a detrimental role in post-stroke inflammation. Suppressing inflammasome activation by targeting the checkpoint executing enzymes Caspase-1/-11 displayed promising therapeutic effects.

## Data Availability Statement

The original contributions presented in the study are included in the article/Supplementary Material, further inquiries can be directed to the corresponding author/s.

## Ethics Statement

The animal study was reviewed and approved by The animal experimental studies were approved by the Animal Care and Use Committee of Sun Yat-Sen University. Written informed consent was obtained from the individual(s) for the publication of any potentially identifiable images or data included in this article.

## Author Contributions

DL designed and performed the experiments, collected and analyzed data, and drafted the manuscript. MH performed animal experiments and collected data. BZ, YL QZ, and XM contributed to the experimental design and revised the manuscript. WC and ZL designed and supervised the study and critically revised the manuscript. All authors read and approved the final manuscript.

## Conflict of Interest

The authors declare that the research was conducted in the absence of any commercial or financial relationships that could be construed as a potential conflict of interest.

## References

[1] Semper RP Vieth M Gerhard M Mejias-Luque R. *Helicobacter pylori* exploits the NLRC4 inflammasome to dampen host defenses. J Immunol. (2019) 203:2183–93. 10.4049/jimmunol.1900351 31511355

[2] Shin JI Lee KH Joo YH Lee JM Jeon J Jung HJ. Inflammasomes and autoimmune and rheumatic diseases: a comprehensive review. J Autoimmun. (2019) 103:102299. 10.1016/j.jaut.2019.06.010 31326231

[3] Barrington J Lemarchand E Allan SM. A brain in flame; do inflammasomes and pyroptosis influence stroke pathology? Brain Pathol. (2017) 27:205–12. 10.1111/bpa.12476 27997059 PMC8028888

[4] Gao L Dong Q Song Z Shen F Shi J Li Y. NLRP3 inflammasome: a promising target in ischemic stroke. Inflamm Res. (2017) 66:17–24. 10.1007/s00011-016-0981-7 27576327

[5] Hong P Gu RN Li FX Xiong XX Liang WB You ZJ. NLRP3 inflammasome as a potential treatment in ischemic stroke concomitant with diabetes. J Neuroinflammation. (2019) 16:121. 10.1186/s12974-019-1498-0 31174550 PMC6554993

[6] Lemarchand E Barrington J Chenery A Haley M Coutts G Allen JE. Extent of ischemic brain injury after thrombotic stroke is independent of the NLRP3 (NACHT, LRR and PYD Domains-Containing Protein 3) inflammasome. Stroke. (2019) 50:1232–9. 10.1161/STROKEAHA.118.023620 31009361 PMC6485300

[7] Hu MY Lin YY Zhang BJ Lu DL Lu ZQ Cai W. Update of inflammasome activation in microglia/macrophage in aging and aging-related disease. CNS Neurosci Ther. (2019) 25:1299–307. 10.1111/cns.13262 31729181 PMC6887669

[8] Broz P Dixit VM. Inflammasomes: mechanism of assembly, regulation and signalling. Nat Rev Immunol. (2016) 16:407–20. 10.1038/nri.2016.58 27291964

[9] Kahlenberg JM Kang I. Advances in disease mechanisms and translational technologies: clinicopathologic significance of inflammasome activation in autoimmune diseases. Arthritis Rheumatol. (2020) 72:386–95. 10.1002/art.41127 31562704 PMC7050400

[10] Zhang CJ Jiang M Zhou H Liu W Wang C Kang Z. TLR-stimulated IRAKM activates caspase-8 inflammasome in microglia and promotes neuroinflammation. J Clin Invest. (2018) 128:5399–412. 10.1172/JCI121901 30372424 PMC6264724

[11] Morozova V Cohen LS Makki AE Shur A Pilar G El Idrissi A. Normal and pathological tau uptake mediated by M1/M3 muscarinic receptors promotes opposite neuronal changes. Front Cell Neurosci. (2019) 13:403. 10.3389/fncel.2019.00403 31555098 PMC6737038

[12] Freeman L Guo H David CN Brickey WJ Jha S Ting JP. NLR members NLRC4 and NLRP3 mediate sterile inflammasome activation in microglia and astrocytes. J Exp Med. (2017) 214:1351–70. 10.1084/jem.20150237 28404595 PMC5413320

[13] Chen Q Yang Y Hou J Shu Q Yin Y Fu W. Increased gene copy number of DEFA1/DEFA3 worsens sepsis by inducing endothelial pyroptosis. Proc Natl Acad Sci USA. (2019) 116:3161–70. 10.1073/pnas.1812947116 30718392 PMC6386704

[14] Cai W Liu S Hu M Huang F Zhu Q Qiu W. Functional dynamics of neutrophils after ischemic stroke. Transl Stroke Res. (2020) 11:108–21. 10.1007/s12975-019-00694-y 30847778 PMC6993940

[15] Fiebach JB Stief JD Ganeshan R Hotter B Ostwaldt AC Nolte CH. Reliability of two diameters method in determining acute infarct size. Validation as new imaging biomarker. PLoS ONE. (2015) 10:e0140065. 10.1371/journal.pone.0140065 26447761 PMC4598169

[16] Stetler RA Cao G Gao Y Zhang F Wang S Weng Z. Hsp27 protects against ischemic brain injury via attenuation of a novel stress-response cascade upstream of mitochondrial cell death signaling. J Neurosci. (2008) 28:13038–55. 10.1523/JNEUROSCI.4407-08.2008 19052195 PMC2614130

[17] Leaf IA Nakagawa S Johnson BG Cha JJ Mittelsteadt K Guckian KM. Pericyte MyD88 and IRAK4 control inflammatory and fibrotic responses to tissue injury. J Clin Invest. (2017) 127:321–34. 10.1172/JCI87532 27869651 PMC5199713

[18] Stack JH Beaumont K Larsen PD Straley KS Henkel GW Randle JC. IL-converting enzyme/caspase-1 inhibitor VX-765 blocks the hypersensitive response to an inflammatory stimulus in monocytes from familial cold autoinflammatory syndrome patients. J Immunol. (2005) 175:2630–4. 10.4049/jimmunol.175.4.2630 16081838

[19] Kobori M Yang Z Gong D Heissmeyer V Zhu H Jung YK. Wedelolactone suppresses LPS-induced caspase-11 expression by directly inhibiting the IKK complex. Cell Death Differ. (2004) 11:123–30. 10.1038/sj.cdd.4401325 14526390

[20] Wannamaker W Davies R Namchuk M Pollard J Ford P Ku G Decker C Charifson P Weber P Germann UA Kuida K Randle JC. (S)-1-((S)-2-{[1-(4-amino-3-chloro-phenyl)-methanoyl]-amino}-3,3-dimethyl-butanoy l)-pyrrolidine-2-carboxylic acid ((2R,3S)-2-ethoxy-5-oxo-tetrahydro-furan-3-yl)-amide (VX-765), an orally available selective interleukin (IL)-converting enzyme/caspase-1 inhibitor, exhibits potent anti-inflammatory activities by inhibiting the release of IL-1beta and IL-18. J Pharmacol Exp Ther. (2007) 321:509–16. 10.1124/jpet.106.111344 17289835

[21] Sellin ME Maslowski KM Maloy KJ Hardt WD. Inflammasomes of the intestinal epithelium. Trends Immunol. (2015) 36:442–50. 10.1016/j.it.2015.06.002 26166583

[22] Liu L Sun B. Neutrophil pyroptosis: new perspectives on sepsis. Cell Mol Life Sci. (2019) 76:2031–42. 10.1007/s00018-019-03060-1 30877336 PMC11105444

[23] Gelderblom M Leypoldt F Steinbach K Behrens D Choe CU Siler DA. Temporal and spatial dynamics of cerebral immune cell accumulation in stroke. Stroke. (2009) 40:1849–57. 10.1161/STROKEAHA.108.534503 19265055

[24] He XF Zeng YX Li G Feng YK Wu C Liang FY. Extracellular ASC exacerbated the recurrent ischemic stroke in an NLRP3-dependent manner. J Cereb Blood Flow Metab. (2020) 40:1048–60. 10.1177/0271678X19856226 31216943 PMC7181081

