# Supplementary material for: Temporal and Spatial Dynamics of Inflammasome Activation After Ischemic Stroke
Source: Front Neurol. 2021 Apr 22;12:621555. doi: 10.3389/fneur.2021.621555 (PMC8104123; doi:10.3389/fneur.2021.621555)
Supplement: Supplementary file 1 [file Data_Sheet_1.docx]

**Supplemental Figures**

**
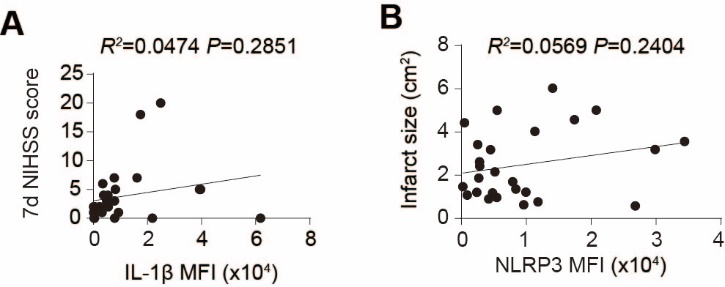
**

**Supplement Figure 1.** (A) Mean fluorescent intensity (MFI) of IL-1β was calculated and correlation of IL-1β MFI with NIHSS score at 7d after disease onset was analyzed with *Linear regression*. (B) MFI of NLRP3 was calculated and the correlation of NLRP3 MFI with infarct size was analyzed with *Linear regression*.

**
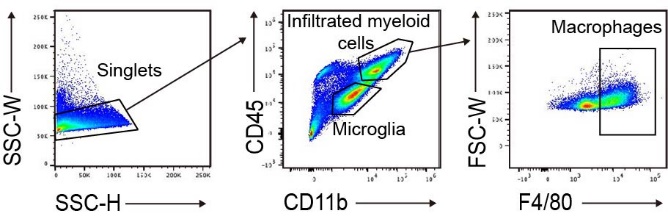
**

**Supplement Figure 2.** The gating strategy of microglia and macrophages.

**
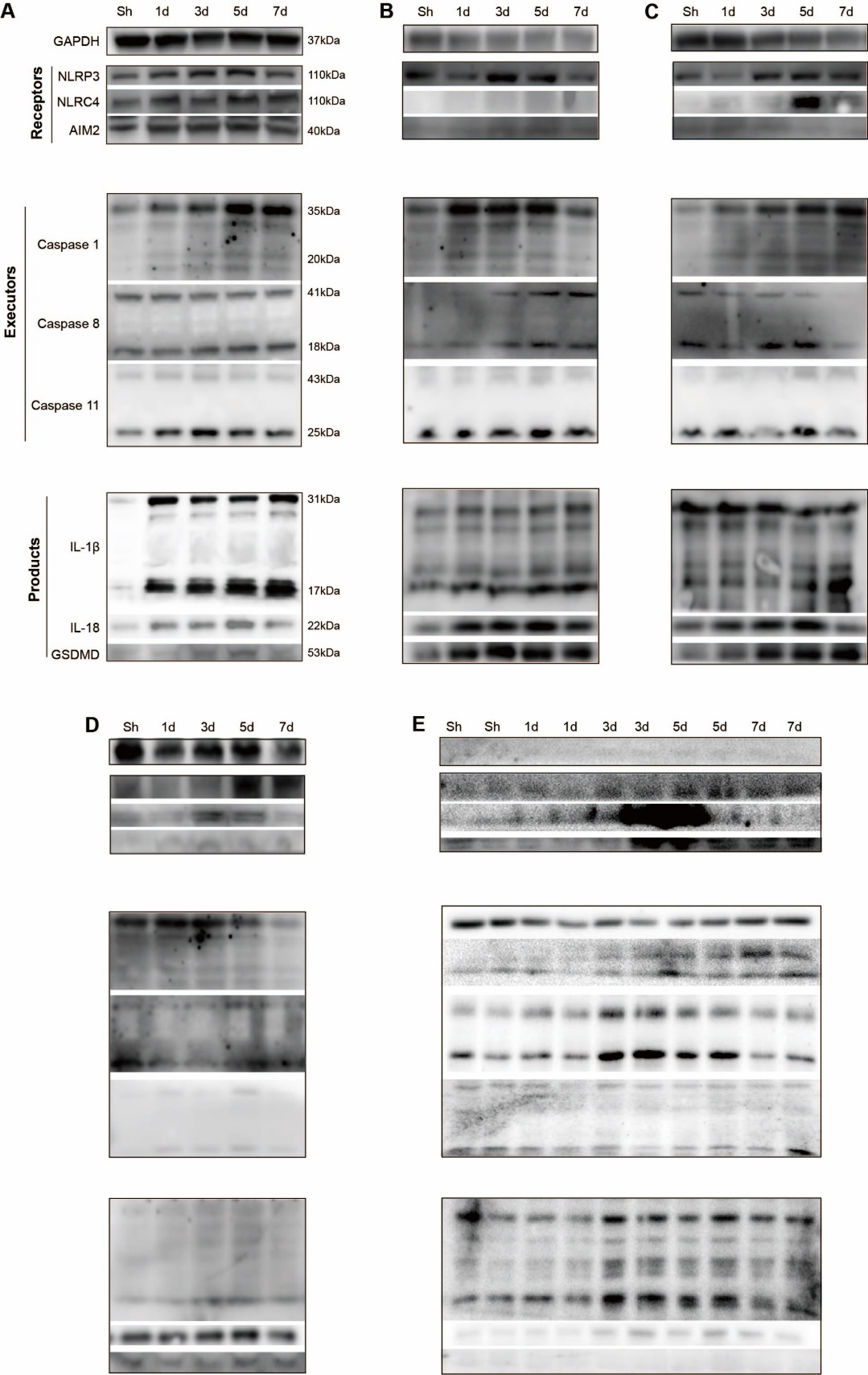
**

**Supplement Figure 3.** The complete western blot images of dynamic alternation of inflammasome after ischemic stroke in mice (n=6).

**Supplement Table 1**

|  | **effect size** | **α** | **total sample size** | **number of groups** | **power** |
| --- | --- | --- | --- | --- | --- |
| NLRP3 | 0.8388 | 0.05 | 30 | 5 | 0.9356 |
| NLRC4 | 0.6474 | 0.05 | 30 | 5 | 0.7363 |
| AIM2 | 0.4878 | 0.05 | 30 | 5 | 0.4654 |
| Caspase1 | 0.5752 | 0.05 | 30 | 5 | 0.6194 |
| Cle-caspase1 | 0.8084 | 0.05 | 30 | 5 | 0.9157 |
| caspase8 | 0.4368 | 0.05 | 30 | 5 | 0.3783 |
| cle-caspase8 | 0.6166 | 0.05 | 30 | 5 | 0.6885 |
| caspase11 | 0.3217 | 0.05 | 30 | 5 | 0.2137 |
| cle-caspase11 | 0.5599 | 0.05 | 30 | 5 | 0.5928 |
| IL-1β | 0.6153 | 0.05 | 30 | 5 | 0.6864 |
| IL-18 | 0.8006 | 0.05 | 30 | 5 | 0.91 |
| GSDMD | 0.7067 | 0.05 | 30 | 5 | 0.8173 |

The power test analysis for dynamic alternation of inflammasome after ischemic stroke in mice.
